# Influence of Artificial Aging of ZnAlCu Alloys on Microstructure and Compressive Yield Strength

**DOI:** 10.3390/ma18214823

**Published:** 2025-10-22

**Authors:** Angelika Kiefel, Alexander Bezold, Christoph Broeckmann

**Affiliations:** Institute for Materials Applications in Mechanical Engineering, 52062 Aachen, Germany; a.bezold@iwm.rwth-aachen.de (A.B.); c.broeckmann@iwm.rwth-aachen.de (C.B.)

**Keywords:** aging, 0.2% compressive yield strength, microstructure

## Abstract

So far, the influence of aging on the mechanical properties of ZnAlCu alloys has primarily been investigated under tensile load. Since some applications, such as plain bearings, are subjected to compressive loads, the results presented in the literature do not fully encompass all areas of application. Therefore, this publication focuses on the influence of artificial aging on the 0.2% compressive yield strength. Samples from ZnAl1Cu0.7, ZnAl11Cu0.7 and ZnAl11Cu2 were aged at different aging temperatures for up to 840 h. After different aging periods, compressive tests as well as microstructure investigations with SEM and XRD were carried out. Furthermore, the dimensional stability of ZnAl11Cu0.7 was investigated in a quenching dilatometer. Shrinkage of up to 0.08%, followed by swelling, was determined. Compressive tests revealed a decrease in the 0.2% compressive yield strength across all tested alloys, most pronounced at the beginning of the aging process, reaching an approximately constant strength level after an alloy- and temperature-dependent aging period. At the end, based on the results, a possible way to determine the constant strength level and the necessary aging time to reach this strength level for specific application temperatures is presented to ensure stable mechanical properties during operation.

## 1. Introduction

Climate change and the associated rethinking of recent decades have led to increased requirements for the recyclability, environmental and health compatibility and cost-effectiveness of bearing materials. In combination with new areas of application, such as use in wind turbines, there is a need for alternative materials.

Due to their exceptional combination of mechanical properties, wear resistance and cost-effectiveness, ZnAlCu alloys are a promising alternative to established plain bearing materials like whites metals or bronzes [1,2,3,4,5,6]. However, as the melting point of ZnAlCu alloys is relatively low, around 400 °C, aging already occurs at room temperature, leading to dimensional changes and a reduction in strength [7,8,9,10,11]. The influence of aging on the mechanical and tribological properties of ZnAlCu alloys has been investigated, among others, by Goodwin et al. [12,13,14,15,16] and Pola et al. [17]. They describe aging as a thermally activated, diffusion-controlled process that gradually alters the microstructure over time and can be expressed by the Arrhenius equation. Over extended periods, these studies have documented a decrease in hardness and tensile strength, as well as microstructural changes [4,9,13,18,19]. For instance, according to Kallien and Leis [4], the tensile strength decreases by up to 15% within one year due to the change in structure, while at the same time the ductility of the aluminum-rich phase increases [20].

**Figure 1:**
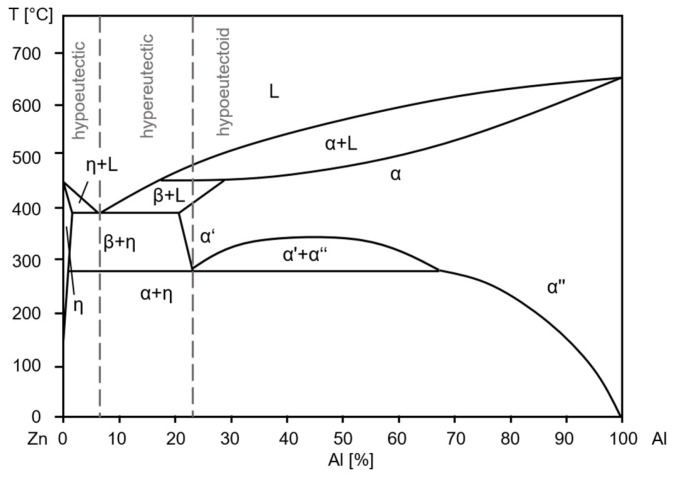
Binary ZnAl phase diagram [26].

The primary cause of aging in these alloys is the presence of metastable high-temperature phases that form during solidification. Since the ZnAlCu alloys investigated in this study have a low copper content, solidification can be explained using the binary ZnAl phase diagram. As depicted in Figure 1, depending on the composition, a distinction is made between hypoeutectic (Al content < 6 wt.%), hypereutectic (Al content between 6 wt.% and 22 wt.%) and hypereutectoid alloys (Al content > 22 wt.%). During the solidification of hypereutectic alloys, the η-phase is the first to separate from the melt. When the temperature drops to around 380 °C, the remaining liquid undergoes a eutectic transformation into the zinc-rich η-phase and the aluminum-rich α′-phase. The η-phase forms a hexagonal (hcp) zinc solid solution, while the α′-phase forms a face-centered cubic (fcc) aluminum solid solution. At around 275 °C, a solid-state transformation occurs, and the previously formed α′-phase decomposes into η and the zinc-poor α″-phase. The α″-phase also has an fcc aluminum crystal structure, but differs from α′ in its chemical composition [21,22,23,24]. The hypereutectic ZnAl alloys, on the other hand, solidify primarily in the aluminum-rich α-phase, followed by eutectic solidification and eutectoid decomposition of the α-phase [22,25].

The described microstructure formation takes place under thermodynamic equilibrium, which is an idealized condition that does not occur in practice. Due to non-equilibrium solidification and segregation, eutectic structures can form at aluminum contents up to 20 wt.% [23]. Since aluminum has very low solubility in zinc at room temperature (≈0.05 wt.%), excess aluminum atoms remain supersaturated in the Zn solid solution after solidification, creating a metastable state [12,27,28].

Over time, these phases transform into stable low-temperature equilibrium phases, dissolving the lamellar structure [17]. The duration of this phase transformation is influenced by several factors, including the cooling conditions, alloy composition, wall thickness, and temperature to which the material is exposed [6,18,29,30,31,32,33,34,35].

With the addition of copper, a copper-rich ε-phase begins to form in the interdendritic regions as soon as the copper content exceeds approximately 2 wt.% [8,36]. This intermetallic ε-phase has a hexagonal crystal structure and the chemical composition CuZn_4_ [37,38]. As the copper content increases, a larger number of CuZn_4_ precipitates form, shifting the dominant strengthening mechanism from solid solution strengthening to precipitation hardening. While this enhances the overall strength of the alloy, it also increases its brittleness and raises the risk of crack formation [39,40].

The metastable ε-phase gradually transforms into the stable τ′-phase, which has been identified as Al_4_Cu_3_Zn [39,41,42], Al_3_Cu_5_Zn_2_ [43] or Al_5_Cu_4_Zn [44]. Depending on the copper content, this four-phase reaction (1) can lead to a volume expansion of up to 4.5% [29,35,45].

(1) $$\alpha +\varepsilon \to \;\eta +\tau^{\prime }$$

The progression of the four-phase reaction during aging was confirmed by Zhu et al. [46,47] and Dorantes Rosales et al. [41] using XRD analyses, initially by the coexistence of the ε-phase and the τ′-phase and finally by the complete disappearance of the ε-phase. The τ′-phase forms in the interdendritic regions and along the boundaries of the aluminum-rich phase [47,48].

This natural aging process can be anticipated by artificial aging, thereby stabilizing the properties. Since zinc alloys are frequently used in automotive engineering, the aging tests conducted by Goodwin et al. [12,13,14,15,16] and Pola et al. [17] refer to the temperature range between −35 °C and 105 °C. The selected temperature and aging duration strongly affect the rate of phase transformations and the associated strength reduction [19,32]. Pola et al. [17] reported that ZnAl15Cu1 shows a faster decrease in tensile strength and an increasing coarsening of the microstructure at higher aging temperatures. This accelerated degradation is attributed to enhanced solid-state diffusion and reduced lattice distortions, which promote dislocation motion [17,49]. Kallien and Goodwin describe similar relationships for hypereutectic alloys [4,12].

However, what all studies have in common is that they examine aging behavior under tensile stress. The impact of aging for applications under compressive stress has not yet been sufficiently investigated. Given that the predominant stress in plain bearings is compressive [50], this study examines the influence of aging on the 0.2% compressive yield strength. In addition, plain bearings are often in operation for several decades, so the properties of the bearing material must remain stable throughout their entire service life. It is therefore essential to understand the aging behavior of ZnAlCu alloys to optimize performance and service life.

Since time-consuming treatments to stabilize material properties are not practical in industrial applications, aging experiments were conducted at temperatures of up to 240 °C. This approach is based on Pola’s aforementioned observation that higher aging temperatures accelerate both the decline in tensile strength and the coarsening of the microstructure. Microstructural changes were analyzed using scanning electron microscopy to provide detailed insights into the aging process under these conditions.

Additionally, the dimensional stability of a ZnAlCu alloy was investigated through an aging test using a quenching dilatometer by recording the change in length.

## 2. Materials and Methods

The three ZnAlCu alloys, ZnAl1Cu0.7, ZnAl11Cu0.7 and ZnAl11Cu2, were cast in a mold casting process, and cylindrical test specimens with a height and diameter of 7 mm each were produced. Aging tests were carried out at 120 °C, 180 °C and 240 °C. Therefore, of each alloy, 114 samples were artificially aged in a furnace from two hours for up to 840 h. For all three alloys and at every aging temperature, six samples were removed from the oven after 2 h, 24 h, 72 h, 168 h, 336 h and 504 h. At the aging temperature of 120 °C, six samples were additionally aged for 840 h. From the six samples, five were used for compressive tests, while the remaining one was used for metallographic characterization. The executed aging tests are summarized in Table 1.

A universal testing machine from ZwickRoell type Z020 (Ulm, Germany) with a maximum force application of 20 kN and a quartz glass extensometer were used for compressive tests at about 25 °C (room temperature), 60 °C, 100 °C and 150 °C. A 3-zone furnace from MTS type 652.01D (Berlin, Germany) was used for the tests at elevated temperatures. For all tests, the traverse speed of the crosshead was 0.014 mm/s. A more detailed description of the experimental procedure can be found in [26].

Microstructure investigations were executed by scanning electron microscopy with a type Jeol-6400 microscope (Freising, Germany).

X-ray diffraction (XRD) was deployed for detection of the phases in ZnAl11Cu2 at aging temperatures of 120 °C and 180 °C after 504 h in comparison with the as-cast condition directly after the casting process. An XRD device from Seifert Analytical X-Ray type 2002 PTS Kristalloflex 760 (XRD Eigenmann GmbH, Schnaittach-Hormersdorf, Germany) with a Cu-Kα-source and a Ni filter was used, and a step width of 0.02° and a sampling rate of 0.1 cps were set.

Furthermore, an aging test was carried out on ZnAl11Cu0.7 in a quenching dilatometer type DIL 805A/D/T (TA Instruments, Eschborn, Germany) at 250 °C for 200 h to analyze the dimensional stability.

## 3. Results

### 3.1. Microstructure

**Figure 2:**
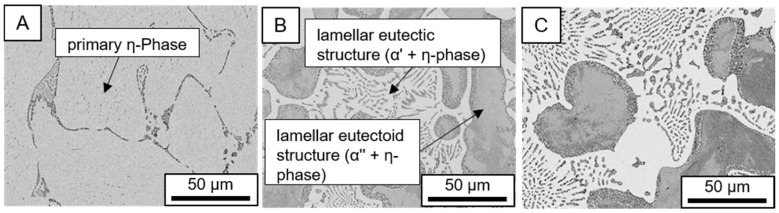
Microstructure after the casting process (as-cast) of (**A**): ZnAl1Cu0.7, (**B**): ZnAl11Cu0.7 and (**C**): ZnAl11Cu2 [26].

The microstructure of the hypoeutectic alloy ZnAl1Cu0.7 is characterized by the primary zinc-rich η-phase, as well as lamellar eutectic and eutectoid structure. The hypereutectic alloys ZnAl11Cu0.7 and ZnAl11Cu2, on the other hand, solidify primarily in the aluminum-rich α-phase, followed by a eutectoid decomposition at 275 °C; the microstructure exhibits exclusively fine lamellar eutectic and eutectoid structures [9,15,51]. The microstructure of ZnAl1Cu0.7, ZnAl11Cu0.7 and ZnAl11Cu2 directly after the casting process (as-cast) is shown in Figure 2. At higher copper contents, the intermetallic ε-phase Zn_4_Cu is formed but could not be detected in the as-cast condition of the investigated alloys. However, since the ε-phase has a comparable material contrast to the η-phase, it is conceivable that the resolution of the SEM was not high enough for detection [32,52].

### 3.2. Dimensional Changes

**Figure 3:**
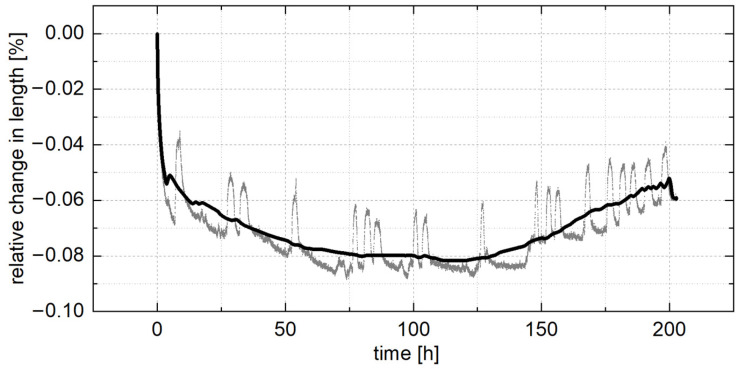
Relative change in length of ZnAl11Cu0.7 during artificial aging in the quenching dilatometer at 250 °C. Grey: original curve; black: fitted curve.

Dimensional changes in ZnAlCu alloys are often described as one of their main disadvantages [29,35]. Therefore, the relative change in length was investigated on the alloy ZnAl11Cu0.7, as shown in Figure 3. It was found that the largest dimensional changes occurred in the first three hours, resulting in a shrinkage of about 0.05%. After 100 h, the shrinkage reached its maximum value of 0.08%, followed by swelling of the sample. Unfortunately, the experiment was interrupted after 200 h due to technical problems and could not be repeated. As described in the literature, the shrinkage at the beginning of the measurement can be attributed to the precipitation of forcibly dissolved aluminum and the following growth to the four-phase reaction [53].

In both cases, the dimensional changes occur due to a change in the lattice parameters. During the precipitation of forcibly dissolved aluminum, the proportion of the aluminum-rich α-phase with a face-centered cubic crystal geometry increases, while the proportion of the zinc-rich η-phase with a hexagonal crystal geometry decreases. Since the face-centered cubic crystal geometry has a smaller lattice constant than the hexagonal crystal geometry, shrinkage occurs. During the four-phase reaction, the metastable copper-rich ε-phase with a hexagonal crystal geometry transforms into the stable τ′-phase with a rhombohedral crystal geometry. Since higher copper contents mean a higher amount of ε-phase, the swelling of the sample increases with increasing copper contents [8,27,53,54].

### 3.3. Microstructural Changes During Aging

The segregation process during aging was investigated in detail using SEM, and the microstructure was determined for each alloy, aging temperature, and aging time. The microstructure is shown in Table 2, Table 3 and Table 4. For all three alloys at 120 °C, no segregation process can be observed over the aging period. At 240 °C, on the other hand, there are aluminum (Al) precipitates after 168 h. This precipitation of aluminum forcibly dissolved in the η-phase primarily takes place in the eutectic phase due to the short diffusion paths between the lamellae, resulting in the decomposition of the eutectic structure [15]. At the aging temperature of 180 °C, only ZnAl11Cu2 shows precipitations after 504 h due to the fact that copper increases the solubility of aluminum in zinc [13].

**Figure 4:**
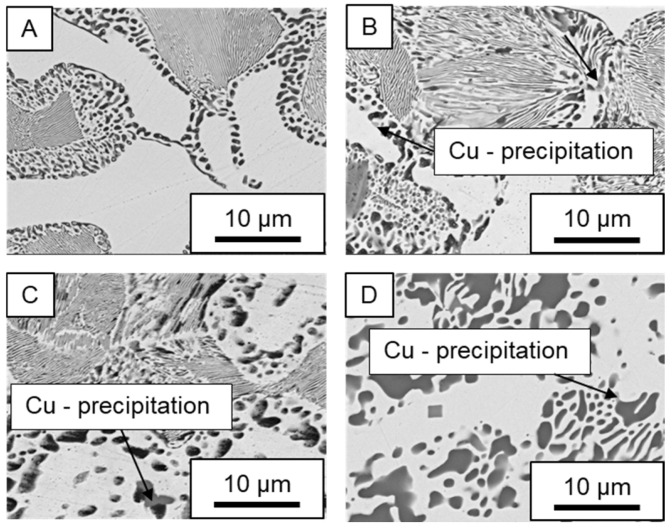
Cu precipitates in ZnAl11Cu0.7 (**A**): as-cast; precipitates after aging at (**B**): 120 °C, (**C**): 180 °C, and (**D**): 240 °C and 504 h [26].

**Figure 5:**
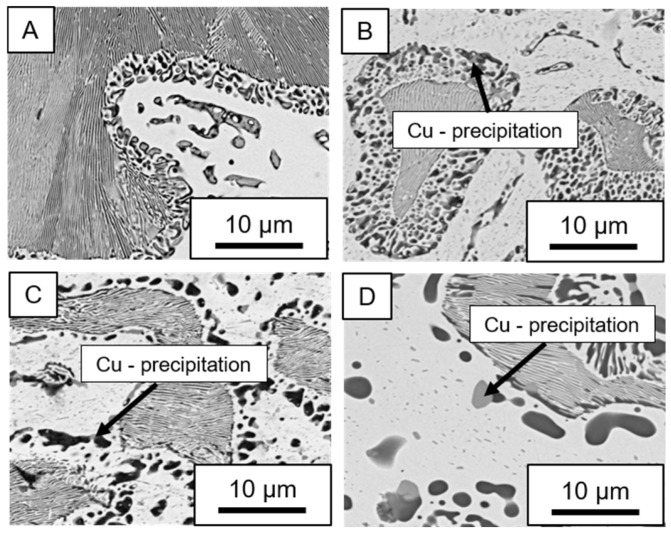
Cu precipitates in ZnAl11Cu2 (**A**): as-cast; precipitates after aging at (**B**): 120 °C, (**C**): 180 °C, and (**D**): 240 °C and 504 h [26].

In ZnAl11Cu0.7 and ZnAl11Cu2, Cu precipitates were observed at all three aging temperatures. Figure 4 shows the copper-rich (Cu) precipitates after 504 h at 120 °C, 180 °C and 240 °C compared to the as-cast condition of ZnAl11Cu0.7. Figure 5 shows the same for ZnAl11Cu2. It is noteworthy that in both alloys, after aging at 120 °C, the Cu precipitates are found in the eutectic structures, whereas after aging at 180 °C and 240 °C, they primarily occur in the η-phase. Furthermore, coarser Cu precipitates are present at higher aging temperatures.

**Figure 6:**
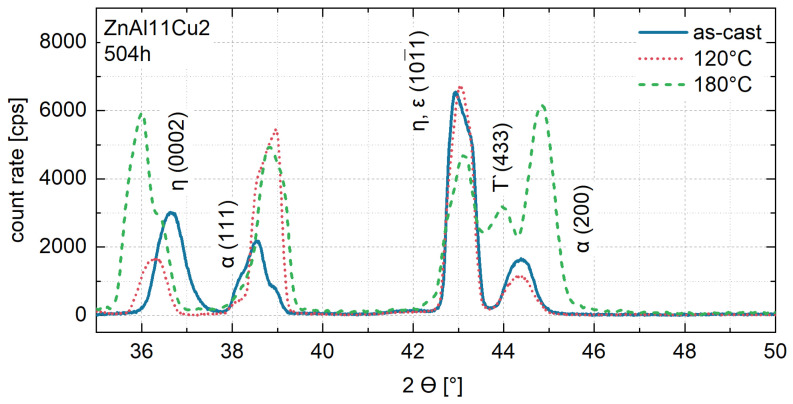
X-ray diffraction measurement of ZnAl11Cu2 after aging for 504 h [26].

The XRD measurements of ZnAl11Cu2 shown in Figure 6 were conducted after 504 h at 120 °C and 180 °C. At both aging temperatures, the η (0002) peak shifts to smaller 2θ angles with increasing aging time. After 504 h at 180 °C, an additional peak appears at 44°. According to Dorantes Rosales et al. [52], this peak corresponds to the τ’-phase resulting from the four-phase reaction. The shift in the η (0002) peak can be explained by the decomposition of the metastable η-phase, from which Al and Cu precipitate. Since the τ′-phase was not detected in the XRD spectrum of ZnAl11Cu2 at 120 °C, the Cu precipitations at 120 °C can be assigned to Zn_4_Cu (ε). After aging at 180 °C, depending on the progress of the four-phase reaction, coexistence of the ε and τ′-phases is likely [46,52].

### 3.4. The 0.2% Compressive Yield Strength

**Figure 7:**
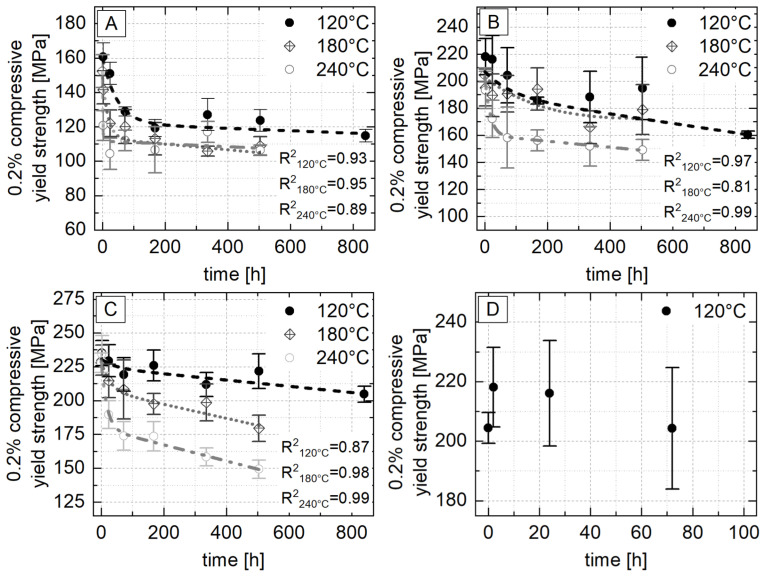
The 0.2% compressive yield strength of (**A**) ZnAl1Cu0.7, (**B**) ZnAl11Cu0.7 and (**C**) ZnAl11Cu2 at 120 °C, 180 °C and 240 °C over the entire aging period, as well as (**D**) ZnAl11Cu0.7 at 120 °C up to 100 h; the dashed lines represent the approximation according to Equation (2) [26].

Figure 7 shows the average values of the 0.2% compressive yield strength obtained from the five compressive tests of each alloy, aging temperature and time. Despite high standard deviations of up to ± 25 MPa, which can be attributed to casting-related inhomogeneities such as segregation, all data can be represented by an exponential function with a linear component of the following form:

(2) $$y=\;p_{1}\;\cdot \;e^{-\frac{x}{p_{2}}}+\;p_{3}+p_{4}\cdot x$$

Although the constant strength level described by Kallien and Leis [4] could not be achieved at any of the three aging temperatures investigated, clear trends can nevertheless be identified that indicate the behavior of the alloy under the given conditions.

For all three alloys, a decrease in strength over the aging time can be observed. The drop in strength is greatest at the beginning of the aging process and decreases with increasing aging time.

A comparison of the 0.2% yield strengths for all three alloys across the aging temperatures investigated shows a consistent decrease in strength with increasing temperature, which is largely independent of the aging duration. The only exception occurs after 168 h, where the yield strength at 180 °C is slightly higher than at 120 °C. However, this difference is within the standard deviation of ±16 MPa and is therefore negligible.

A closer look at the initial aging phase in Figure 7D shows that at 120 °C, the average 0.2% compressive yield strength increases by about 7% within the first 2 h compared to the as-cast condition, before gradually falling back to its original level after 72 h. This short-term increase in strength, which is also reported by Savaskan and Murphy [35], is not statistically significant due to high standard deviations and does not occur at 180 °C or 240 °C.

**Figure 8:**
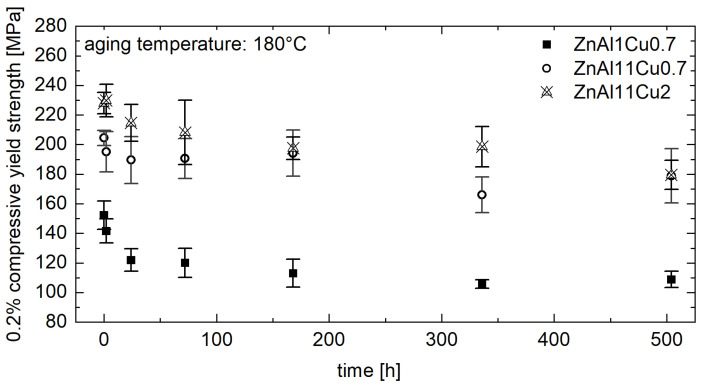
The 0.2% yield strength of ZnAl1Cu0.7, ZnAl11Cu0.7 and ZnAl11Cu2 measured at RT after aging at 180 °C [26].

Figure 8 shows that the 0.2% compressive yield strength increases with increasing alloy contents, regardless of the aging temperature and time. This can be attributed to solid solution strengthening [3].

**Figure 9:**
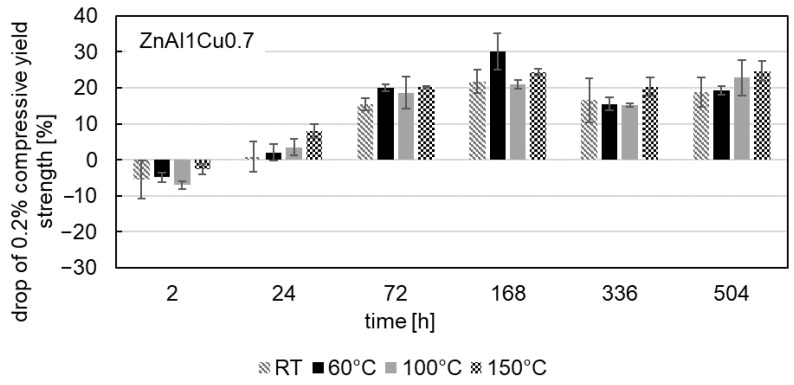
Drop in 0.2% compressive yield strength during aging at 120 °C of ZnAl1Cu0.7 for compressive test temperatures of RT, 60 °C, 100 °C and 150 °C.

**Figure 10:**
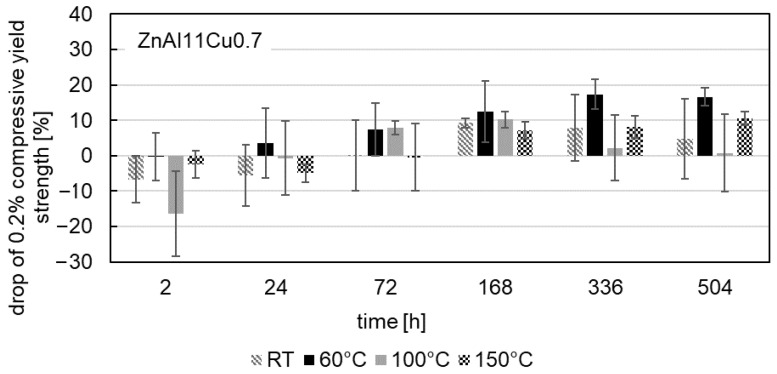
Drop in 0.2% compressive yield strength during aging at 120 °C of ZnAl11Cu0.7 for compressive test temperatures of RT, 60 °C, 100 °C and 150 °C.

**Figure 11:**
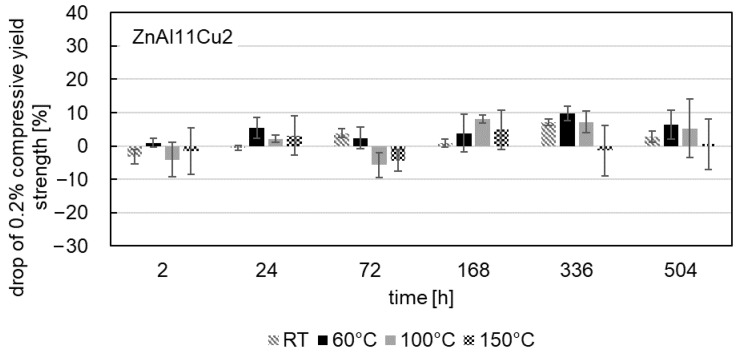
Drop in 0.2% compressive yield strength during aging at 120 °C of ZnAl11Cu2 for compressive test temperatures of RT, 60 °C, 100 °C and 150 °C.

The influence of aging on the 0.2% compressive yield strength was also investigated at test temperatures of 60 °C, 100 °C and 150 °C after aging at a temperature of 120 °C. Figure 9, Figure 10 and Figure 11 show the drop in 0.2% compressive yield strength for all three alloys. The aging process at test temperatures of 60 °C, 100 °C and 150 °C is comparable to that at RT. At the beginning of the aging process, an increase in the 0.2% compressive yield strength can be observed, followed by a drop in strength.

The maximum drop in strength occurs in ZnAl11Cu0.7 at a test temperature of 100 °C after 2 h. Regardless of the aging time, the lowest 0.2% compressive yield strengths are observed at high test temperatures (highest drop in strength). The cause of this phenomenon is the increased energy input at higher temperatures, which enhances dislocation movement. Consequently, greater plastic deformation occurs under the same force.

## 4. Discussion

### 4.1. Correlation Between Microstructure and 0.2% Compressive Yield Strength During Aging

**Figure 12:**
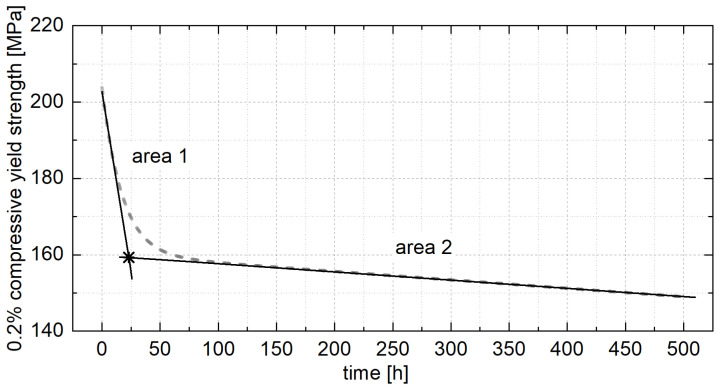
Schematic diagram of the intersection of the fitted straight lines from areas 1 and 2 [26].

The aging time required to reach an approximately constant strength level can be determined by dividing the curve into two areas, as shown in Figure 12. The area at the beginning of aging shows a sharp drop in the 0.2% compressive yield strength, and the area of aging shows an approximately constant 0.2% compressive yield strength. A similar behavior was observed by Kallien and Leis [4] for ZnAl4Cu1 at RT, where area 2 reached a constant strength level after about 270 days. Therefore, an approximately constant strength level can be assumed after a certain aging period.

Based on this assumption and Equation (2), two linear fits are approximated for areas 1 and 2 to determine the duration of artificial aging required to achieve an approximately constant strength level and the 0.2% yield strength that describes this strength level. However, it must be considered that even if the assumption of a linear function at the beginning of aging may be a good approximation for determining the point in time at which the constant strength level is reached, it cannot fully describe the strength reduction. Particularly at low aging temperatures, an increase in the 0.2% compressive yield strength can initially be observed for all alloys before the strength begins to decrease.

**Figure 13:**
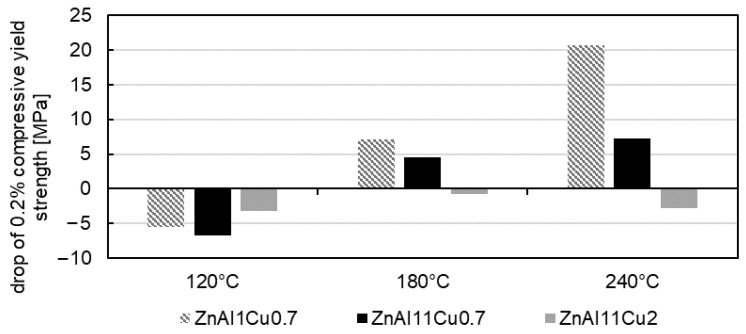
Drop in 0.2% compressive yield strength in the first 2 h of aging [26].

Due to earlier results from the literature [17,35], the drop in the 0.2% compressive yield strength during aging can be attributed to segregation processes in which Al is precipitated from the zinc-rich η-phase. Figure 13 shows the drop in 0.2% compressive yield strength in the first 2 h of aging. Depending on the alloy and aging temperature, an increase of up to 7% can be observed. This increase in strength is attributed to nucleation, while the following decrease results from grain growth [35]. At 120 °C, all three alloys show this progression. At higher temperatures, however, due to the higher diffusion rate within the solid solution at elevated temperatures, no initial increase in 0.2% compressive yield strength is observed for ZnAl1Cu0.7 and ZnAl11Cu0.7. This leads to an overlap of nucleation and grain growth processes [35]. Aging experiments conducted for less than 2 h would likely have shown an initial increase in the 0.2% compressive yield strength, but these were not investigated in detail. An exception to this behavior is observed in the ZnAl11Cu2 alloy. At all three aging temperatures, an initial increase in the 0.2% compressive yield strength occurred. This is due to copper reducing the decomposition rate of the α′-phase, resulting in a delay of the transformation process [35].

No clear correlation could be observed between microstructural changes and the decrease in the 0.2% compressive yield strength. It can therefore be assumed that the decrease in strength is not solely attributable to segregation processes and the loss of solid solution strengthening, but that other mechanisms also contribute to the decrease in strength.

Table 5 summarizes the drop in 0.2% compressive yield strength in area 1, the decrease in strength per hour in areas 1 und 2, the aging time until the stress level is reached, and the 0.2% compressive yield strength at the approximately constant stress level. Higher aluminum contents lead to a higher amount of forcibly dissolved aluminum in the η-phase. Therefore, a higher drop in strength was expected for the alloys with 11 wt.% aluminum than for the alloy with 1 wt.% aluminum, accompanied by longer aging times [12,55,56]. However, the highest drop in strength was observed for ZnAl1Cu0.7, with 26% at 240 °C and a decrease in strength of 15.81 MPa/h. One possible explanation for this behavior could be the incomplete aging process, as only an approximately constant strength level was achieved. This is supported by the fact that the decrease in strength in area 2 is greater for the alloys with 11 wt.% aluminum than for the alloy with 1 wt.% aluminum.

The time required to reach an approximately constant strength level varies depending on the alloy composition and the aging temperature. For ZnAl1Cu0.7, for example, the time required to reach the strength level at 120 °C is 87 h, whereas at 240 °C, the strength level is already reached after 3 h. The influence of the copper content is also clearly reflected in the results. The four-phase reaction, which increases with rising copper contents, also contributes to the decrease in strength and leads to shorter aging times until an approximately constant strength level is reached [41,46,47]. At 180 °C, the strength reduction of ZnAl11Cu2 at 0.58 MPa/h in area 1 is about three times higher than that of ZnAl11Cu0.7 at 0.18 MPa/h.

A closer look at the influence of aging temperature on strength shows that hypereutectic alloys in particular exhibit a greater drop in strength as the aging temperature increases. The loss of strength of ZnAl11Cu2 is 3% at 120 °C until an approximately constant strength level is reached, but 21% at 240 °C. However, the influence of the aging temperature on the drop in strength is lower for ZnAl1Cu0.7. This observation could be related to the segregation processes described by Goodwin et al. [12] and Mykura et al. [55], which could not be detected in the present work using SEM. The coarsening of the microstructure is also cited in the literature as a cause of strength loss [35,41,57] but was not investigated in this work.

### 4.2. Constant Strength Level and Aging Time for Unknown Temperatures

Since the constant strength level correlates with the aging temperature, the aging temperature must be selected in relation to the operating temperature of the target application. The following section outlines a method to determine aging temperatures and times based on the results in order to achieve a constant strength level. This approach assumes that the approximately constant strength level is a constant one. To obtain more accurate results, additional tests with longer aging times are necessary. Furthermore, experiments by Goodwin and Kallien [16] have shown that the influence of sample size on aging cannot be neglected. A dimensional factor that enables us to transfer the results to sample geometries other than those used in this study has not yet been investigated. Nevertheless, a possible approach for ZnAl11Cu2 is described below, which also works for the other two alloys investigated.

**Figure 14:**
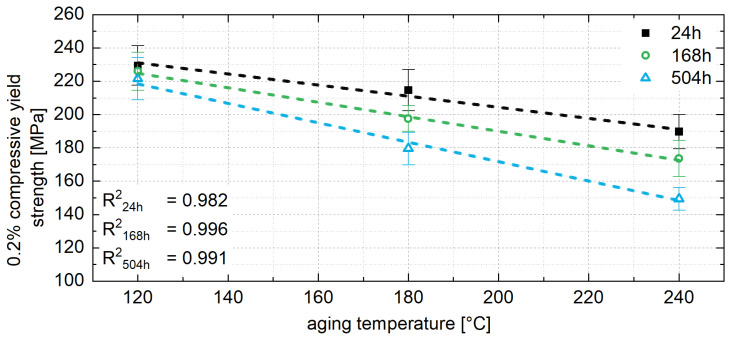
Relationship between 0.2% compressive yield strength and aging temperature for ZnAl11Cu2 after 22 h, 168 h and 504 h [26].

**Figure 15:**
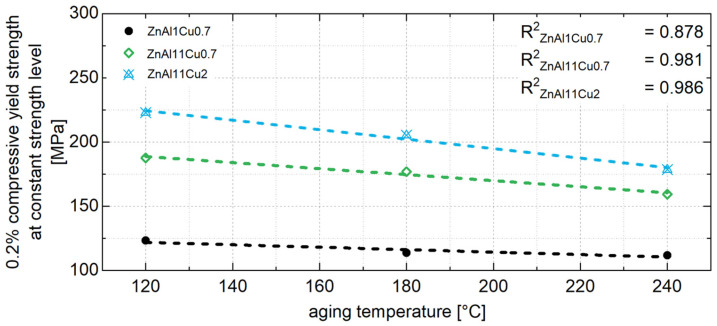
Relationship between 0.2% compressive yield strength at the approximately constant stress level and aging temperature for ZnAl1Cu0.7, ZnAl11Cu0.7 and ZnAl11Cu2 [26].

As shown in Figure 14 for ZnAl11Cu2, a linear relationship can be determined between the 0.2% compressive yield strength and the aging temperature. Since the relationship shown in Figure 14 can be observed at any time during aging, it is assumed that the influence of the aging temperature on strength can also be linearly correlated for other aging temperatures and times. This linear relationship includes the correlation between constant stress level and aging temperature, as shown in Figure 15.

**Figure 16:**
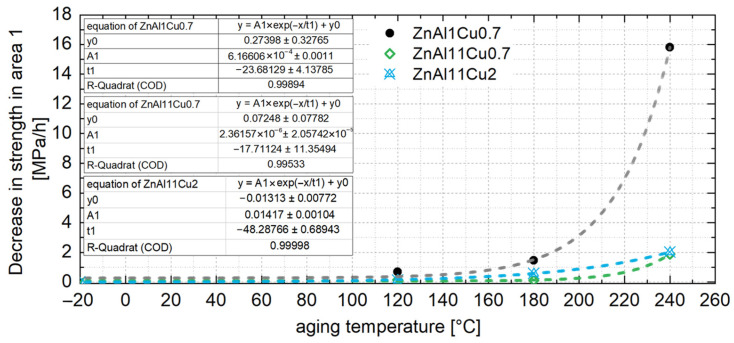
Decrease in strength of ZnAl1Cu0.7, ZnAl11Cu0.7 and ZnAl11Cu2 in area 1 of the aging process as a function of the aging temperature; the absolute values are plotted [26].

From the linear relationship between the 0.2% compressive yield strength and aging temperature, the constant strength level for untested temperatures can be interpolated. This allows the constant strength level for a specific operating temperature to be estimated. Aging the material to this strength level prior to use ensures that the mechanical properties remain stable during operation. The aging time required to achieve this constant strength level can be determined from the exponential relationship between the decrease in strength in area 1 and the aging temperature, as shown in Figure 16 for all three alloys. Since no aging and no drop in strength are expected below 0.4 times of the absolute melting temperature, Figure 16 includes a data point at −18 °C, which corresponds to the storage temperature of the samples prior to the aging tests.

If the drop in strength in area 1 is described by a linear function, the aging time x until a constant strength level y is reached can be determined using Equation (3), where m represents the drop in strength in area 1, and n is the 0.2% compressive yield strength in the as-cast condition.

(3) $$x=\frac{y-n}{m}$$

This approach allows the constant strength level and the aging time required to achieve this constant strength level at unknown temperatures to be determined, ensuring reliable performance of the material in practical applications.

## 5. Conclusions

This study investigated the dimensional changes and mechanical properties during aging of the three ZnAlCu alloys ZnAl1Cu0.7, ZnAl11Cu0.7 and ZnAl11Cu2. The results can be summarized as follows:

- The largest dimensional changes occurred in the first three hours, resulting in shrinkage of approximately 0.05%, with a maximum shrinkage value of 0.08% after 100 h, followed by length increases. The dimensional changes during aging were related to changes in the lattice parameters due to aluminum precipitation and the four-phase reaction.
- Microstructural analysis with SEM showed no segregation at the aging temperature of 120 °C. At 240 °C, aluminum precipitations were detected after 168 h, and after 504 h, copper precipitations were observed for all three alloys. XRD measurements of ZnAl11Cu2 at 120 °C and 180 °C indicated phase transformations involving the η (0002) peak and the appearance of the τ′-phase.
- Mechanical tests revealed a consistent decrease in the 0.2% compressive yield strength for all alloys tested over the aging time, which was most pronounced at the beginning of the aging process. With progressive aging, an approximately constant strength level was adjusted.
- In contrast to the decrease in strength, an increase was observed for ZnAl1Cu0.7 and ZnAl11Cu0.7 in the first 2 h. This increase can be attributed to nucleation followed by grain growth, which leads to the observed loss of strength. However, this increase was not observed in ZnAl11Cu2, which is due to a reduced decomposition rate of the α′-phase, resulting in a delay of the transformation process.
- A higher approximately constant strength level was achieved with higher aluminum and copper contents due to solid solution strengthening.
- Increased aging temperatures led to faster aging processes and shorter times to reach the constant stress level, which could be related to segregation but was not detected in the SEM in the present study.
- No clear correlation between microstructural changes and the decrease in 0.2% compressive yield strength could be determined.
- To ensure stable mechanical properties during operation, it is essential to age the material until the constant strength level is reached. A linear relationship between the 0.2% compressive yield strength and aging temperature was observed and used to interpolate the constant strength level for a specific operating temperature. The corresponding aging time was determined from the exponential relationship between the decrease in strength at the beginning of aging and the aging temperature.

These results presented provide a framework for determining aging temperatures and times to achieve a constant strength level, thereby enhancing the reliability of ZnAlCu alloys in engineering applications.

This article is based on the author’s doctoral dissertation, published by Shaker Verlag, Düren, 2025 [26].

## Figures and Tables

**Table 1:** Aging tests carried out on ZnAl1Cu0.7, ZnAl11Cu0.7 and ZnAl11Cu2 [26].

	2 h	24 h	72 h	168 h	336 h	504 h	840 h
120 °C	x	x	x	x	x	x	x
180 °C	x	x	x	x	x	x	
240 °C	x	x	x	x	x	x	

**Table 2:** Microstructure development during aging of ZnAl1Cu0.7 [26].

	24 h	168 h	504 h
120 °C	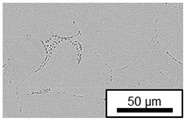	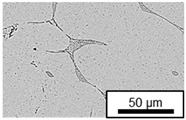	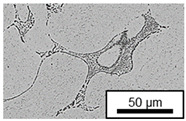
180 °C	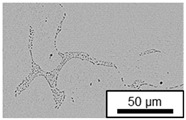	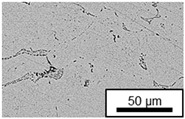	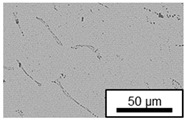
240 °C	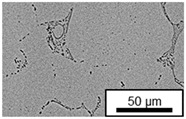	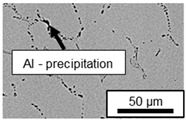	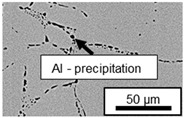

**Table 3:** Microstructure development during aging of ZnAl11Cu0.7 [26].

	24 h	168 h	504 h
120 °C	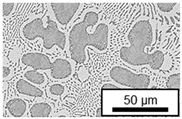	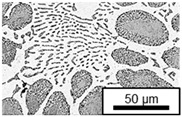	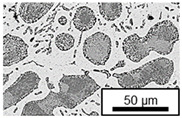
180 °C	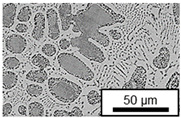	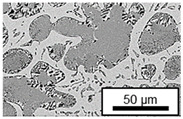	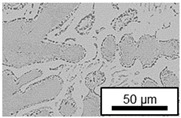
240 °C	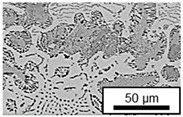	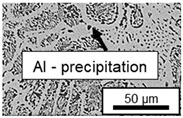	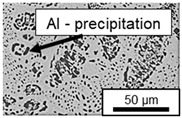

**Table 4:** Microstructure development during aging of ZnAl11Cu2 [26].

	24 h	168 h	504 h
120 °C	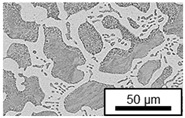	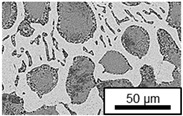	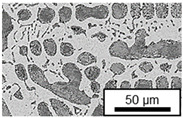
180 °C	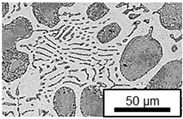	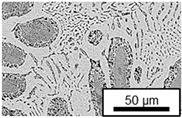	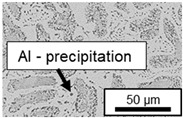
240 °C	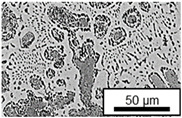	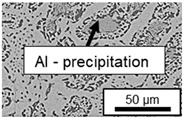	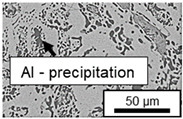

**Table 5:** Comparison of aging time and drop in strength of ZnAl1Cu0.7, ZnAl11Cu0.7 and ZnAl11Cu2 until reaching the approximately constant strength level (area 1) and the drop in strength in the range of the approximately constant strength level (area 2) assuming two linear functions [26].

Alloy	Aging Temperature [°C]	Drop in Strength in Area 1 [%]	Decrease in Strength in Area 1 [MPa/h]	Decrease in Strength in Area 2 [MPa/h]	Aging Time Until Constant Stress Level is Reached [h]	0.2% Compressive Yield Strength at Constant Stress Level [MPa]
ZnAl1Cu0.7	120	19	0.42	9.23 × 10^−3^	87	123
	180	25	1.08	1.87 × 10^−2^	32	114
	240	26	15.81	8.16 × 10^−3^	3	112
ZnAl11Cu0.7	120	9	0.12	3.80 × 10^−2^	168	186
	180	13	0.18	1.63 × 10^−2^	139	178
	240	22	1.33	2.15 × 10^−2^	33	159
ZnAl11Cu2	120	3	0.15	2.28 × 10^−2^	58	222
	180	10	0.58	5.36 × 10^−2^	39	206
	240	21	2.03	6.17 × 10^−2^	25	179

## Data Availability

The original contributions presented in this study are included in the article. Further inquiries can be directed to the corresponding author.

## Abbreviations

The following abbreviations are used in this manuscript:

Al	aluminum
Cu	copper
RT	room temperature
SEM	scanning electron microscopy
XRD	X-ray diffraction
Zn	zinc
